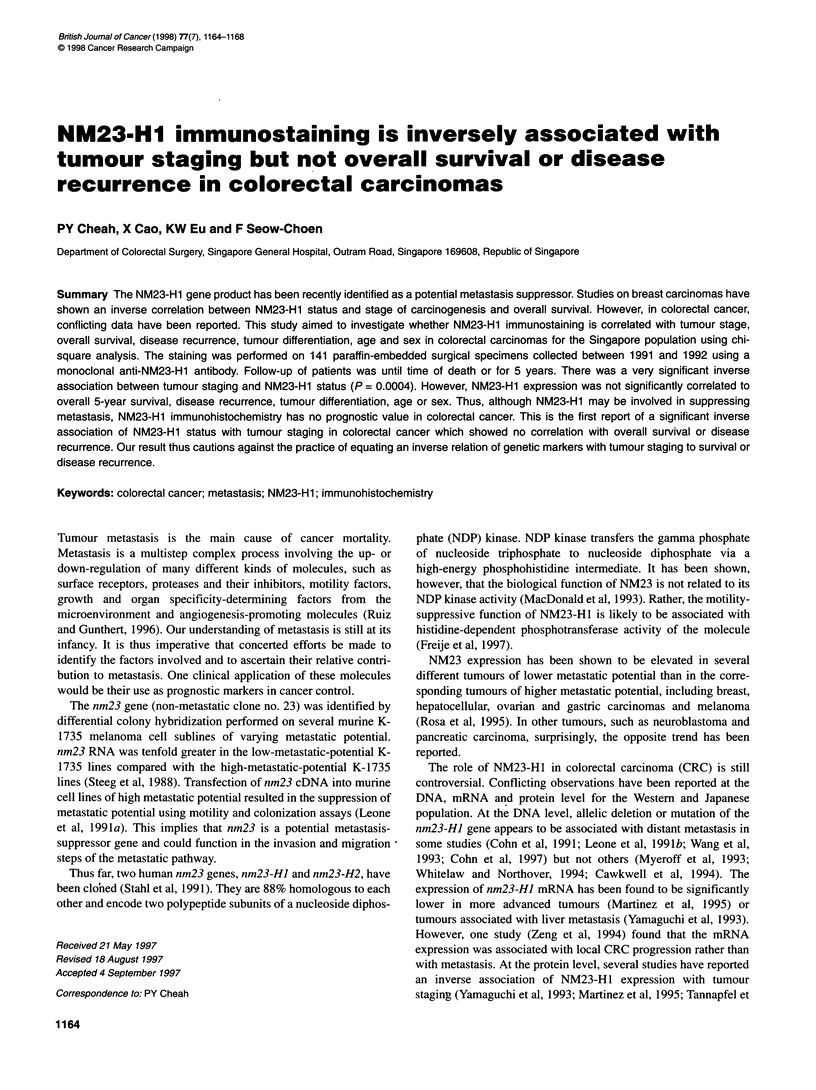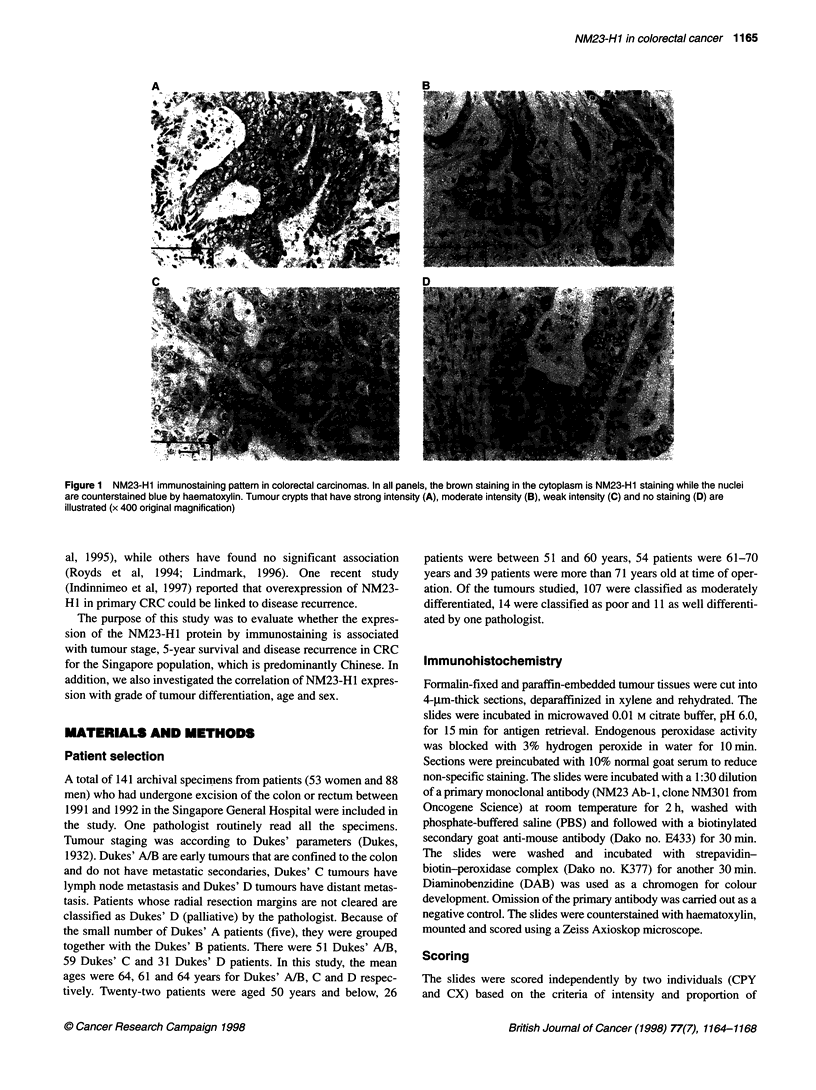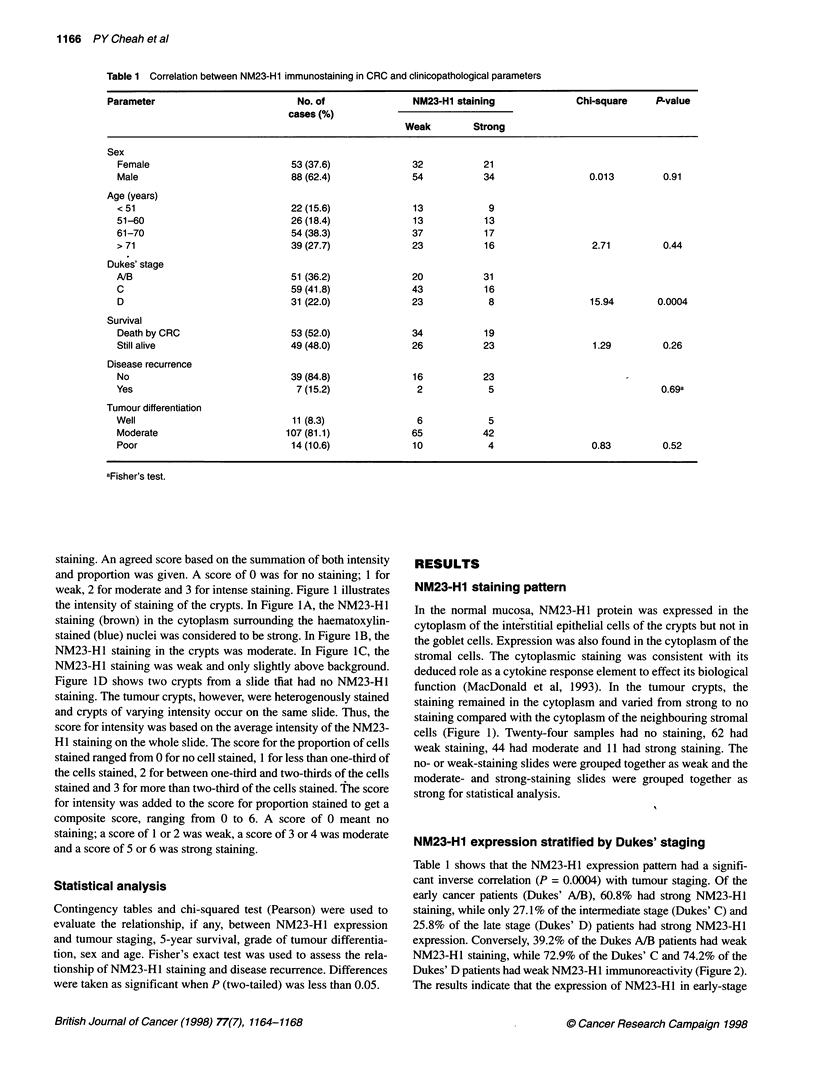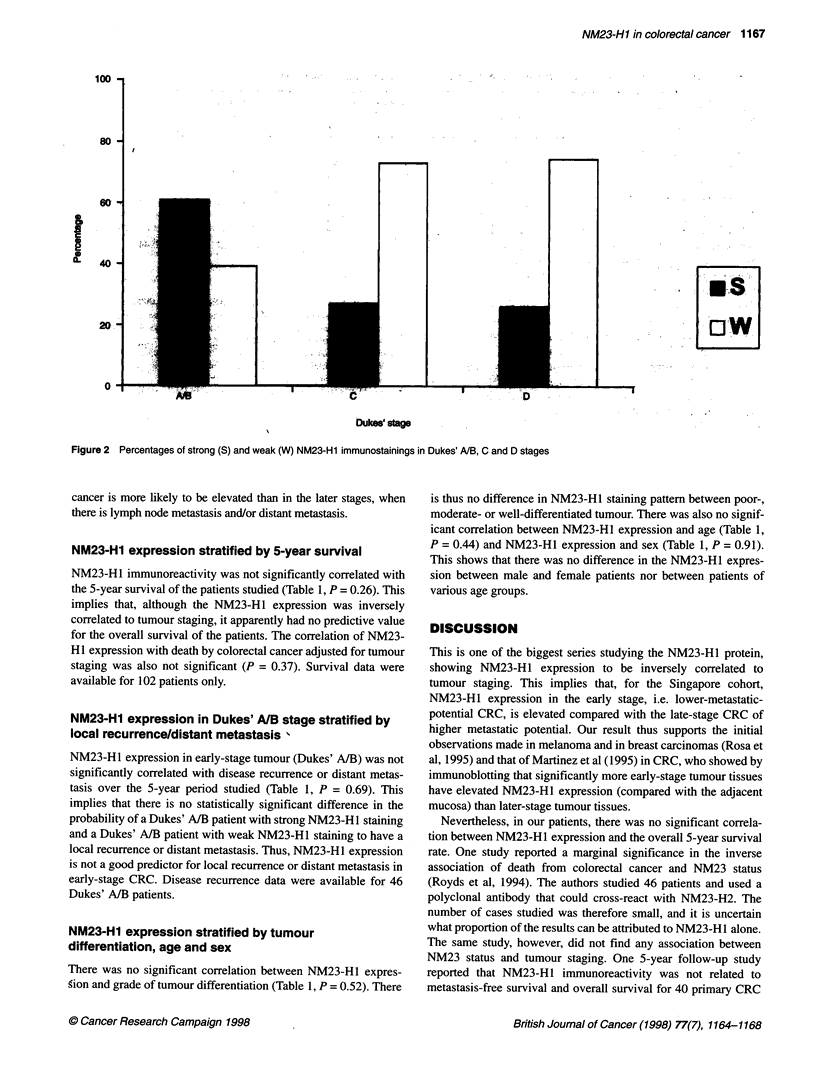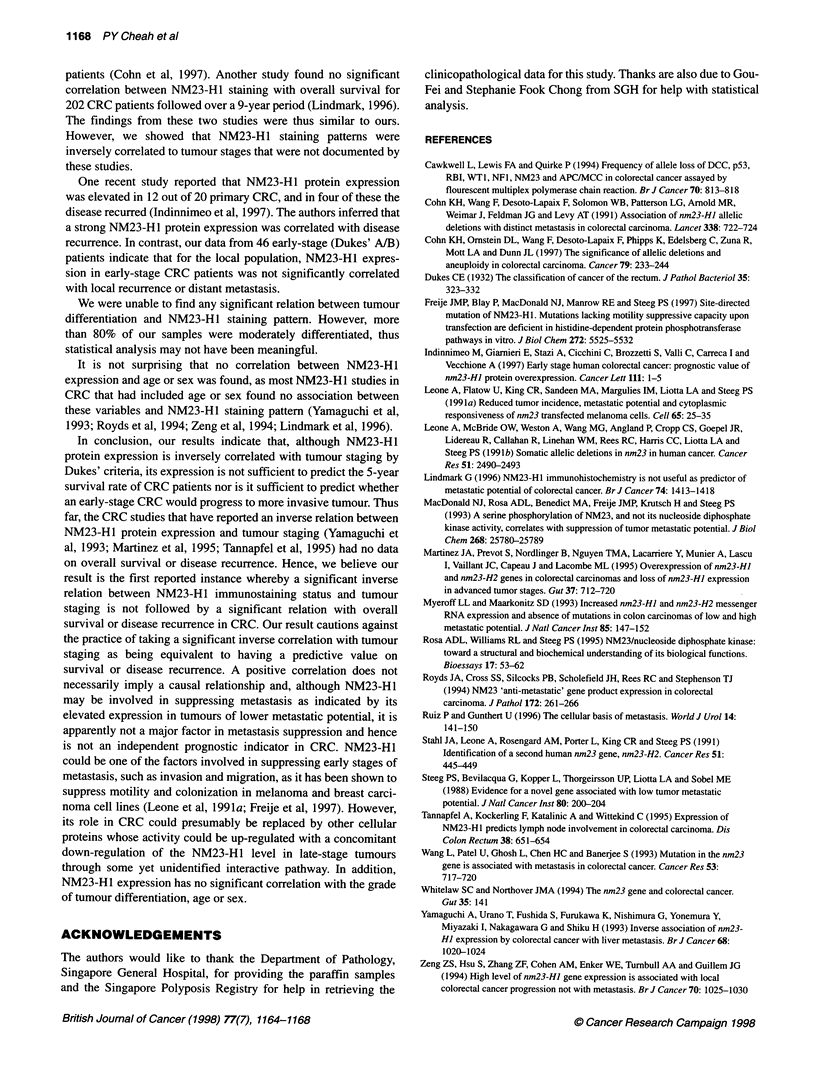# NM23-H1 immunostaining is inversely associated with tumour staging but not overall survival or disease recurrence in colorectal carcinomas.

**DOI:** 10.1038/bjc.1998.193

**Published:** 1998-04

**Authors:** P. Y. Cheah, X. Cao, K. W. Eu, F. Seow-Choen

**Affiliations:** Department of Colorectal Surgery, Singapore General Hospital, Republic of Singapore.

## Abstract

**Images:**


British Journal of Cancer (1998) 77(7), 1164–1168
© 1998 Cancer Research Campaign

### NM23-H1 immunostaining is inversely associated with tumour staging but not overall survival or disease recurrence in colorectal carcinomas

**PY Cheah, X Cao, KW Eu and F Seow-Choen**

Department of Colorectal Surgery, Singapore General Hospital, Outram Road, Singapore 169608, Republic of Singapore

**Summary** The NM23-H1 gene product has been recently identified as a potential metastasis suppressor. Studies on breast carcinomas have shown an inverse correlation between NM23-H1 status and stage of carcinogenesis and overall survival. However, in colorectal cancer, conflicting data have been reported. This study aimed to investigate whether NM23-H1 immunostaining is correlated with tumour stage, overall survival, disease recurrence, tumour differentiation, age and sex in colorectal carcinomas for the Singapore population using chi-square analysis. The staining was performed on 141 paraffin-embedded surgical specimens collected between 1991 and 1992 using a monoclonal anti-NM23-H1 antibody. Follow-up of patients was until time of death or for 5 years. There was a very significant inverse association between tumour staging and NM23-H1 status ($P = 0.0004$). However, NM23-H1 expression was not significantly correlated to overall 5-year survival, disease recurrence, tumour differentiation, age or sex. Thus, although NM23-H1 may be involved in suppressing metastasis, NM23-H1 immunohistochemistry has no prognostic value in colorectal cancer. This is the first report of a significant inverse association of NM23-H1 status with tumour staging in colorectal cancer which showed no correlation with overall survival or disease recurrence. Our result thus cautions against the practice of equating an inverse relation of genetic markers with tumour staging to survival or disease recurrence.

**Keywords:** colorectal cancer; metastasis; NM23-H1; immunohistochemistry

Tumour metastasis is the main cause of cancer mortality. Metastasis is a multistep complex process involving the up- or down-regulation of many different kinds of molecules, such as surface receptors, proteases and their inhibitors, motility factors, growth and organ specificity-determining factors from the microenvironment and angiogenesis-promoting molecules (Ruiz and Gunthert, 1996). Our understanding of metastasis is still at its infancy. It is thus imperative that concerted efforts be made to identify the factors involved and to ascertain their relative contribution to metastasis. One clinical application of these molecules would be their use as prognostic markers in cancer control.

The *nm23* gene (non-metastatic clone no. 23) was identified by differential colony hybridization performed on several murine K-1735 melanoma cell sublines of varying metastatic potential. *nm23* RNA was tenfold greater in the low-metastatic-potential K-1735 lines compared with the high-metastatic-potential K-1735 lines (Steeg et al, 1988). Transfection of *nm23* cDNA into murine cell lines of high metastatic potential resulted in the suppression of metastatic potential using motility and colonization assays (Leone et al, 1991*a*). This implies that *nm23* is a potential metastasis-suppressor gene and could function in the invasion and migration steps of the metastatic pathway.

Thus far, two human *nm23* genes, *nm23-H1* and *nm23-H2*, have been cloned (Stahl et al, 1991). They are 88% homologous to each other and encode two polypeptide subunits of a nucleoside diphosphate (NDP) kinase. NDP kinase transfers the gamma phosphate of nucleoside triphosphate to nucleoside diphosphate via a high-energy phosphohistidine intermediate. It has been shown, however, that the biological function of NM23 is not related to its NDP kinase activity (MacDonald et al, 1993). Rather, the motility-suppressive function of NM23-H1 is likely to be associated with histidine-dependent phosphotransferase activity of the molecule (Freije et al, 1997).

NM23 expression has been shown to be elevated in several different tumours of lower metastatic potential than in the corresponding tumours of higher metastatic potential, including breast, hepatocellular, ovarian and gastric carcinomas and melanoma (Rosa et al, 1995). In other tumours, such as neuroblastoma and pancreatic carcinoma, surprisingly, the opposite trend has been reported.

The role of NM23-H1 in colorectal carcinoma (CRC) is still controversial. Conflicting observations have been reported at the DNA, mRNA and protein level for the Western and Japanese population. At the DNA level, allelic deletion or mutation of the *nm23-H1* gene appears to be associated with distant metastasis in some studies (Cohn et al, 1991; Leone et al, 1991*b*; Wang et al, 1993; Cohn et al, 1997) but not others (Myeroff et al, 1993; Whitelaw and Northover, 1994; Cawkwell et al, 1994). The expression of *nm23-H1* mRNA has been found to be significantly lower in more advanced tumours (Martinez et al, 1995) or tumours associated with liver metastasis (Yamaguchi et al, 1993). However, one study (Zeng et al, 1994) found that the mRNA expression was associated with local CRC progression rather than with metastasis. At the protein level, several studies have reported an inverse association of NM23-H1 expression with tumour staging (Yamaguchi et al, 1993; Martinez et al, 1995; Tannapfel et

*Received 21 May 1997*
*Revised 18 August 1997*
*Accepted 4 September 1997*

*Correspondence to:* PY Cheah

**Figure 1** NM23-H1 immunostaining pattern in colorectal carcinomas. In all panels, the brown staining in the cytoplasm is NM23-H1 staining while the nuclei are counterstained blue by haematoxylin. Tumour crypts that have strong intensity (**A**), moderate intensity (**B**), weak intensity (**C**) and no staining (**D**) are illustrated (× 400 original magnification)

al, 1995), while others have found no significant association (Royds et al, 1994; Lindmark, 1996). One recent study (Indinnimeo et al, 1997) reported that overexpression of NM23-H1 in primary CRC could be linked to disease recurrence.

The purpose of this study was to evaluate whether the expression of the NM23-H1 protein by immunostaining is associated with tumour stage, 5-year survival and disease recurrence in CRC for the Singapore population, which is predominantly Chinese. In addition, we also investigated the correlation of NM23-H1 expression with grade of tumour differentiation, age and sex.

### MATERIALS AND METHODS

### Patient selection

A total of 141 archival specimens from patients (53 women and 88 men) who had undergone excision of the colon or rectum between 1991 and 1992 in the Singapore General Hospital were included in the study. One pathologist routinely read all the specimens. Tumour staging was according to Dukes' parameters (Dukes, 1932). Dukes' A/B are early tumours that are confined to the colon and do not have metastatic secondaries, Dukes' C tumours have lymph node metastasis and Dukes' D tumours have distant metastasis. Patients whose radial resection margins are not cleared are classified as Dukes' D (palliative) by the pathologist. Because of the small number of Dukes' A patients (five), they were grouped together with the Dukes' B patients. There were 51 Dukes' A/B, 59 Dukes' C and 31 Dukes' D patients. In this study, the mean ages were 64, 61 and 64 years for Dukes' A/B, C and D respectively. Twenty-two patients were aged 50 years and below, 26 patients were between 51 and 60 years, 54 patients were 61–70 years and 39 patients were more than 71 years old at time of operation. Of the tumours studied, 107 were classified as moderately differentiated, 14 were classified as poor and 11 as well differentiated by one pathologist.

### Immunohistochemistry

Formalin-fixed and paraffin-embedded tumour tissues were cut into 4-μm-thick sections, deparaffinized in xylene and rehydrated. The slides were incubated in microwaved 0.01 M citrate buffer, pH 6.0, for 15 min for antigen retrieval. Endogenous peroxidase activity was blocked with 3% hydrogen peroxide in water for 10 min. Sections were preincubated with 10% normal goat serum to reduce non-specific staining. The slides were incubated with a 1:30 dilution of a primary monoclonal antibody (NM23 Ab-1, clone NM301 from Oncogene Science) at room temperature for 2 h, washed with phosphate-buffered saline (PBS) and followed with a biotinylated secondary goat anti-mouse antibody (Dako no. E433) for 30 min. The slides were washed and incubated with strepavidin–biotin–peroxidase complex (Dako no. K377) for another 30 min. Diaminobenzidine (DAB) was used as a chromogen for colour development. Omission of the primary antibody was carried out as a negative control. The slides were counterstained with haematoxylin, mounted and scored using a Zeiss Axioskop microscope.

### Scoring

The slides were scored independently by two individuals (CPY and CX) based on the criteria of intensity and proportion of

© Cancer Research Campaign 1998

**Table 1** Correlation between NM23-H1 immunostaining in CRC and clinicopathological parameters

| Parameter | No. of cases (%) | NM23-H1 staining | | Chi-square | P-value |
|---|---|---|---|---|---|
| | | Weak | Strong | | |
| Sex | | | | | |
| Female | 53 (37.6) | 32 | 21 | | |
| Male | 88 (62.4) | 54 | 34 | 0.013 | 0.91 |
| Age (years) | | | | | |
| < 51 | 22 (15.6) | 13 | 9 | | |
| 51–60 | 26 (18.4) | 13 | 13 | | |
| 61–70 | 54 (38.3) | 37 | 17 | | |
| > 71 | 39 (27.7) | 23 | 16 | 2.71 | 0.44 |
| Dukes' stage | | | | | |
| A/B | 51 (36.2) | 20 | 31 | | |
| C | 59 (41.8) | 43 | 16 | | |
| D | 31 (22.0) | 23 | 8 | 15.94 | 0.0004 |
| Survival | | | | | |
| Death by CRC | 53 (52.0) | 34 | 19 | | |
| Still alive | 49 (48.0) | 26 | 23 | 1.29 | 0.26 |
| Disease recurrence | | | | | |
| No | 39 (84.8) | 16 | 23 | | |
| Yes | 7 (15.2) | 2 | 5 | | 0.69[a] |
| Tumour differentiation | | | | | |
| Well | 11 (8.3) | 6 | 5 | | |
| Moderate | 107 (81.1) | 65 | 42 | | |
| Poor | 14 (10.6) | 10 | 4 | 0.83 | 0.52 |

[a]Fisher's test.

staining. An agreed score based on the summation of both intensity and proportion was given. A score of 0 was for no staining; 1 for weak, 2 for moderate and 3 for intense staining. Figure 1 illustrates the intensity of staining of the crypts. In Figure 1A, the NM23-H1 staining (brown) in the cytoplasm surrounding the haematoxylin-stained (blue) nuclei was considered to be strong. In Figure 1B, the NM23-H1 staining in the crypts was moderate. In Figure 1C, the NM23-H1 staining was weak and only slightly above background. Figure 1D shows two crypts from a slide that had no NM23-H1 staining. The tumour crypts, however, were heterogenously stained and crypts of varying intensity occur on the same slide. Thus, the score for intensity was based on the average intensity of the NM23-H1 staining on the whole slide. The score for the proportion of cells stained ranged from 0 for no cell stained, 1 for less than one-third of the cells stained, 2 for between one-third and two-thirds of the cells stained and 3 for more than two-third of the cells stained. The score for intensity was added to the score for proportion stained to get a composite score, ranging from 0 to 6. A score of 0 meant no staining; a score of 1 or 2 was weak, a score of 3 or 4 was moderate and a score of 5 or 6 was strong staining.

### Statistical analysis

Contingency tables and chi-squared test (Pearson) were used to evaluate the relationship, if any, between NM23-H1 expression and tumour staging, 5-year survival, grade of tumour differentiation, sex and age. Fisher's exact test was used to assess the relationship of NM23-H1 staining and disease recurrence. Differences were taken as significant when $P$ (two-tailed) was less than 0.05.

### RESULTS

### NM23-H1 staining pattern

In the normal mucosa, NM23-H1 protein was expressed in the cytoplasm of the interstitial epithelial cells of the crypts but not in the goblet cells. Expression was also found in the cytoplasm of the stromal cells. The cytoplasmic staining was consistent with its deduced role as a cytokine response element to effect its biological function (MacDonald et al, 1993). In the tumour crypts, the staining remained in the cytoplasm and varied from strong to no staining compared with the cytoplasm of the neighbouring stromal cells (Figure 1). Twenty-four samples had no staining, 62 had weak staining, 44 had moderate and 11 had strong staining. The no- or weak-staining slides were grouped together as weak and the moderate- and strong-staining slides were grouped together as strong for statistical analysis.

### NM23-H1 expression stratified by Dukes' staging

Table 1 shows that the NM23-H1 expression pattern had a significant inverse correlation ($P$ = 0.0004) with tumour staging. Of the early cancer patients (Dukes' A/B), 60.8% had strong NM23-H1 staining, while only 27.1% of the intermediate stage (Dukes' C) and 25.8% of the late stage (Dukes' D) patients had strong NM23-H1 expression. Conversely, 39.2% of the Dukes A/B patients had weak NM23-H1 staining, while 72.9% of the Dukes' C and 74.2% of the Dukes' D patients had weak NM23-H1 immunoreactivity (Figure 2). The results indicate that the expression of NM23-H1 in early-stage

© Cancer Research Campaign 1998

**Figure 2** Percentages of strong (S) and weak (W) NM23-H1 immunostainings in Dukes' A/B, C and D stages cancer is more likely to be elevated than in the later stages, when there is lymph node metastasis and/or distant metastasis.

### NM23-H1 expression stratified by 5-year survival

NM23-H1 immunoreactivity was not significantly correlated with the 5-year survival of the patients studied (Table 1, $P = 0.26$). This implies that, although the NM23-H1 expression was inversely correlated to tumour staging, it apparently had no predictive value for the overall survival of the patients. The correlation of NM23-H1 expression with death by colorectal cancer adjusted for tumour staging was also not significant ($P = 0.37$). Survival data were available for 102 patients only.

### NM23-H1 expression in Dukes' A/B stage stratified by local recurrence/distant metastasis

NM23-H1 expression in early-stage tumour (Dukes' A/B) was not significantly correlated with disease recurrence or distant metastasis over the 5-year period studied (Table 1, $P = 0.69$). This implies that there is no statistically significant difference in the probability of a Dukes' A/B patient with strong NM23-H1 staining and a Dukes' A/B patient with weak NM23-H1 staining to have a local recurrence or distant metastasis. Thus, NM23-H1 expression is not a good predictor for local recurrence or distant metastasis in early-stage CRC. Disease recurrence data were available for 46 Dukes' A/B patients.

### NM23-H1 expression stratified by tumour differentiation, age and sex

There was no significant correlation between NM23-H1 expression and grade of tumour differentiation (Table 1, $P = 0.52$). There is thus no difference in NM23-H1 staining pattern between poor-, moderate- or well-differentiated tumour. There was also no significant correlation between NM23-H1 expression and age (Table 1, $P = 0.44$) and NM23-H1 expression and sex (Table 1, $P = 0.91$). This shows that there was no difference in the NM23-H1 expression between male and female patients nor between patients of various age groups.

### DISCUSSION

This is one of the biggest series studying the NM23-H1 protein, showing NM23-H1 expression to be inversely correlated to tumour staging. This implies that, for the Singapore cohort, NM23-H1 expression in the early stage, i.e. lower-metastatic-potential CRC, is elevated compared with the late-stage CRC of higher metastatic potential. Our result thus supports the initial observations made in melanoma and in breast carcinomas (Rosa et al, 1995) and that of Martinez et al (1995) in CRC, who showed by immunoblotting that significantly more early-stage tumour tissues have elevated NM23-H1 expression (compared with the adjacent mucosa) than later-stage tumour tissues.

Nevertheless, in our patients, there was no significant correlation between NM23-H1 expression and the overall 5-year survival rate. One study reported a marginal significance in the inverse association of death from colorectal cancer and NM23 status (Royds et al, 1994). The authors studied 46 patients and used a polyclonal antibody that could cross-react with NM23-H2. The number of cases studied was therefore small, and it is uncertain what proportion of the results can be attributed to NM23-H1 alone. The same study, however, did not find any association between NM23 status and tumour staging. One 5-year follow-up study reported that NM23-H1 immunoreactivity was not related to metastasis-free survival and overall survival for 40 primary CRC

© Cancer Research Campaign 1998

patients (Cohn et al, 1997). Another study found no significant correlation between NM23-H1 staining with overall survival for 202 CRC patients followed over a 9-year period (Lindmark, 1996). The findings from these two studies were thus similar to ours. However, we showed that NM23-H1 staining patterns were inversely correlated to tumour stages that were not documented by these studies.

One recent study reported that NM23-H1 protein expression was elevated in 12 out of 20 primary CRC, and in four of these the disease recurred (Indinnimeo et al, 1997). The authors inferred that a strong NM23-H1 protein expression was correlated with disease recurrence. In contrast, our data from 46 early-stage (Dukes' A/B) patients indicate that for the local population, NM23-H1 expression in early-stage CRC patients was not significantly correlated with local recurrence or distant metastasis.

We were unable to find any significant relation between tumour differentiation and NM23-H1 staining pattern. However, more than 80% of our samples were moderately differentiated, thus statistical analysis may not have been meaningful.

It is not surprising that no correlation between NM23-H1 expression and age or sex was found, as most NM23-H1 studies in CRC that had included age or sex found no association between these variables and NM23-H1 staining pattern (Yamaguchi et al, 1993; Royds et al, 1994; Zeng et al, 1994; Lindmark et al, 1996).

In conclusion, our results indicate that, although NM23-H1 protein expression is inversely correlated with tumour staging by Dukes' criteria, its expression is not sufficient to predict the 5-year survival rate of CRC patients nor is it sufficient to predict whether an early-stage CRC would progress to more invasive tumour. Thus far, the CRC studies that have reported an inverse relation between NM23-H1 protein expression and tumour staging (Yamaguchi et al, 1993; Martinez et al, 1995; Tannapfel et al, 1995) had no data on overall survival or disease recurrence. Hence, we believe our result is the first reported instance whereby a significant inverse relation between NM23-H1 immunostaining status and tumour staging is not followed by a significant relation with overall survival or disease recurrence in CRC. Our result cautions against the practice of taking a significant inverse correlation with tumour staging as being equivalent to having a predictive value on survival or disease recurrence. A positive correlation does not necessarily imply a causal relationship and, although NM23-H1 may be involved in suppressing metastasis as indicated by its elevated expression in tumours of lower metastatic potential, it is apparently not a major factor in metastasis suppression and hence is not an independent prognostic indicator in CRC. NM23-H1 could be one of the factors involved in suppressing early stages of metastasis, such as invasion and migration, as it has been shown to suppress motility and colonization in melanoma and breast carcinoma cell lines (Leone et al, 1991*a*; Freije et al, 1997). However, its role in CRC could presumably be replaced by other cellular proteins whose activity could be up-regulated with a concomitant down-regulation of the NM23-H1 level in late-stage tumours through some yet unidentified interactive pathway. In addition, NM23-H1 expression has no significant correlation with the grade of tumour differentiation, age or sex.

### ACKNOWLEDGEMENTS

The authors would like to thank the Department of Pathology, Singapore General Hospital, for providing the paraffin samples and the Singapore Polyposis Registry for help in retrieving the clinicopathological data for this study. Thanks are also due to Gou-Fei and Stephanie Fook Chong from SGH for help with statistical analysis.

### REFERENCES

Cawkwell L, Lewis FA and Quirke P (1994) Frequency of allele loss of DCC, p53, RBI, WT1, NF1, NM23 and APC/MCC in colorectal cancer assayed by flourescent multiplex polymerase chain reaction. *Br J Cancer* **70**: 813–818

Cohn KH, Wang F, Desoto-Lapaix F, Solomon WB, Patterson LG, Arnold MR, Weimar J, Feldman JG and Levy AT (1991) Association of *nm23-H1* allelic deletions with distinct metastasis in colorectal carcinoma. *Lancet* **338**: 722–724

Cohn KH, Ornstein DL, Wang F, Desoto-Lapaix F, Phipps K, Edelsberg C, Zuna R, Mott LA and Dunn JL (1997) The significance of allelic deletions and aneuploidy in colorectal carcinoma. *Cancer* **79**: 233–244

Dukes CE (1932) The classification of cancer of the rectum. *J Pathol Bacteriol* **35**: 323–332

Freije JMP, Blay P, MacDonald NJ, Manrow RE and Steeg PS (1997) Site-directed mutation of NM23-H1. Mutations lacking motility suppressive capacity upon transfection are deficient in histidine-dependent protein phosphotransferase pathways in vitro. *J Biol Chem* **272**: 5525–5532

Indinnimeo M, Giarnieri E, Stazi A, Cicchini C, Brozzetti S, Valli C, Carreca I and Vecchione A (1997) Early stage human colorectal cancer: prognostic value of *nm23-H1* protein overexpression. *Cancer Lett* **111**: 1–5

Leone A, Flatow U, King CR, Sandeen MA, Margulies IM, Liotta LA and Steeg PS (1991*a*) Reduced tumor incidence, metastatic potential and cytoplasmic responsiveness of *nm23* transfected melanoma cells. *Cell* **65**: 25–35

Leone A, McBride OW, Weston A, Wang MG, Angland P, Cropp CS, Goepel JR, Lidereau R, Callahan R, Linehan WM, Rees RC, Harris CC, Liotta LA and Steeg PS (1991*b*) Somatic allelic deletions in *nm23* in human cancer. *Cancer Res* **51**: 2490–2493

Lindmark G (1996) NM23-H1 immunohistochemistry is not useful as predictor of metastatic potential of colorectal cancer. *Br J Cancer* **74**: 1413–1418

MacDonald NJ, Rosa ADL, Benedict MA, Freije JMP, Krutsch H and Steeg PS (1993) A serine phosphorylation of NM23, and not its nucleoside diphosphate kinase activity, correlates with suppression of tumor metastatic potential. *J Biol Chem* **268**: 25780–25789

Martinez JA, Prevot S, Nordlinger B, Nguyen TMA, Lacarriere Y, Munier A, Lascu I, Vaillant JC, Capeau J and Lacombe ML (1995) Overexpression of *nm23-H1* and *nm23-H2* genes in colorectal carcinomas and loss of *nm23-H1* expression in advanced tumor stages. *Gut* **37**: 712–720

Myeroff LL and Maarkonitz SD (1993) Increased *nm23-H1* and *nm23-H2* messenger RNA expression and absence of mutations in colon carcinomas of low and high metastatic potential. *J Natl Cancer Inst* **85**: 147–152

Rosa ADL, Williams RL and Steeg PS (1995) NM23/nucleoside diphosphate kinase: toward a structural and biochemical understanding of its biological functions. *Bioessays* **17**: 53–62

Royds JA, Cross SS, Silcocks PB, Scholefield JH, Rees RC and Stephenson TJ (1994) NM23 'anti-metastatic' gene product expression in colorectal carcinoma. *J Pathol* **172**: 261–266

Ruiz P and Gunthert U (1996) The cellular basis of metastasis. *World J Urol* **14**: 141–150

Stahl JA, Leone A, Rosengard AM, Porter L, King CR and Steeg PS (1991) Identification of a second human *nm23* gene, *nm23-H2*. *Cancer Res* **51**: 445–449

Steeg PS, Bevilacqua G, Kopper L, Thorgeirsson UP, Liotta LA and Sobel ME (1988) Evidence for a novel gene associated with low tumor metastatic potential. *J Natl Cancer Inst* **80**: 200–204

Tannapfel A, Kockerling F, Katalinic A and Wittekind C (1995) Expression of NM23-H1 predicts lymph node involvement in colorectal carcinoma. *Dis Colon Rectum* **38**: 651–654

Wang L, Patel U, Ghosh L, Chen HC and Banerjee S (1993) Mutation in the *nm23* gene is associated with metastasis in colorectal cancer. *Cancer Res* **53**: 717–720

Whitelaw SC and Northover JMA (1994) The *nm23* gene and colorectal cancer. *Gut* **35**: 141

Yamaguchi A, Urano T, Fushida S, Furukawa K, Nishimura G, Yonemura Y, Miyazaki I, Nakagawara G and Shiku H (1993) Inverse association of *nm23-H1* expression by colorectal cancer with liver metastasis. *Br J Cancer* **68**: 1020–1024

Zeng ZS, Hsu S, Zhang ZF, Cohen AM, Enker WE, Turnbull AA and Guillem JG (1994) High level of *nm23-H1* gene expression is associated with local colorectal cancer progression not with metastasis. *Br J Cancer* **70**: 1025–1030

© Cancer Research Campaign 1998